# Effects of the Irregular Shelterwood System on Regeneration Dynamics of *Shorea robusta* Gaertn. f. in Baijalpur Community Forest, Nepal

**DOI:** 10.1002/ece3.72885

**Published:** 2026-01-04

**Authors:** Sudhan Gaire, Sandesh Gharti, Rohit Bhusal, Badri Bhattarai, Sachin Timilsina, Dipendra Dhungana

**Affiliations:** ^1^ Institute of Forestry Tribhuvan University Hetauda, Makawanpur Nepal; ^2^ Faculty of Forestry Agriculture and Forestry University Hetauda, Makawanpur Nepal; ^3^ Department of Food and Resource Economics University of Copenhagen Frederiksberg C Denmark; ^4^ AgroParisTech‐Centre Palaiseau France; ^5^ College of Forestry, Agriculture, and Natural Resources University of Arkansas at Monticello Monticello Arkansas USA

**Keywords:** community forest, crown cover, organic matter, silviculture system

## Abstract

The Irregular Shelterwood System (ISS) has recently been introduced in Nepalese community forests to promote sustainable regeneration of *Shorea robusta* (Sal). However, limited research exists on its ecological impacts and regeneration dynamics. This study examined regeneration patterns, soil properties, and environmental factors influencing *S. robusta* regeneration under the ISS in Baijalpur Community Forest, Kapilvastu District, Nepal. Using a stratified systematic random sampling design, data were collected from treated and untreated plots. Results showed a significantly higher density of seedlings (16,800 ha^−1^) and saplings (4693 ha^−1^) in treated plots compared to untreated areas (11,960 and 2688 ha^−1^, respectively), indicating the effectiveness of ISS in enhancing regeneration. Most regeneration originated from seedlings rather than coppice shoots. Soil assessments revealed slightly acidic to neutral sandy loam soils, with higher organic matter (OM) and improved nutrient profiles in treated plots. Crown cover and organic carbon were negatively correlated with regeneration, suggesting that moderate canopy openings favor *S. robusta* establishment. These findings underscore the ecological importance of adaptive silvicultural practices for improving regeneration success, forest resilience, and sustainable community forestry in Nepal.

## Graphical abstract

This study evaluates the effectiveness of the Irregular Shelterwood System (ISS) in promoting the regeneration of *Shorea robusta* (Sal) in Nepal's Baijalpur Community Forest. Findings show that treated areas under ISS had significantly higher seedling and sapling densities, improved soil quality, and better regeneration conditions compared to untreated areas, highlighting ISS as a viable strategy for sustainable forest management.

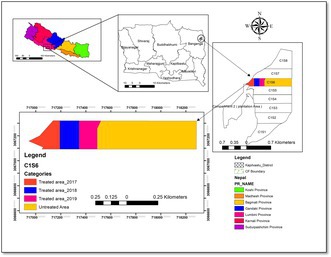

## 1 Introduction

Forest degradation caused by overexploitation and unsustainable harvesting poses a serious threat to forest health in tropical and subtropical regions (Leuschner and Homeier 2022; Gould et al. 2024). Disasters like the earthquake in 2015 also led a decline in sustainable management and usage pattern of forest products within community forests (CFs) in Nepal (Poudel et al. 2022). Sustainable forest management (SFM) is therefore crucial for maintaining biodiversity, enhancing carbon sequestration, and sustaining ecosystem services to recover from catastrophic events like earthquakes and floods, ultimately supporting human livelihoods (Brockerhoff et al. 2017; Fraser 2019; Fichtner and Härdtle 2021; Poudel et al. 2022; Latterini et al. 2023). Effective regeneration of native tree species is a central challenge, particularly in community‐managed forests where local practices and ecological conditions jointly determine forest sustainability (Suoheimo 1999; Chazdon and Guariguata 2016; Löf et al. 2019; Laudari et al. 2024; Timilsina et al. 2024).

*Shorea robusta* Gaertn. f. (Sal) is one of the most ecologically and economically important timber species in South Asia. It is a light‐demanding, deciduous to semi‐deciduous tree species (depending on local climate and site conditions) that forms gregarious stands under suitable conditions (Basyal et al. 2011; Paneru and Chalise 2022; Tinya et al. 2019). In Nepal, *S. robusta* occupies about 15.27% of the total forest area (DFRS 2015) and is valued for its durable timber, resin, and cultural significance (Webb and Sah 2003; Gautam and Devoe 2006; Kumar and Saikia 2020). *S. robusta* timber is widely used in construction due to its strength and longevity (Aryal et al. 2022). Despite its dominance, regeneration failure of *S. robusta* has been a persistent problem in CFs, often attributed to dense canopy cover, grazing pressure, invasive species, and inconsistent management practices (Aryal et al. 2023; Chapagain et al. 2021; Belbase et al. 2023). Regeneration is vital for maintaining forest composition and structure (Khumbongmayum et al. 2005; Liira et al. 2011; Malik and Bhatt 2016). Successful regeneration of *S. robusta* depends on adequate light availability, soil nutrients, and appropriate silvicultural interventions (Osman 2013; Zhu et al. 2014; Sharma et al. 2019). Silvicultural systems such as thinning, pruning, and controlled canopy openings can facilitate natural regeneration by improving microclimatic conditions for seed germination and seedling growth (Löf et al. 2019; Aryal et al. 2023; Belbase et al. 2023).

The Irregular Shelterwood System (ISS) has recently been applied in Nepal to enhance the natural regeneration of *S. robusta* . Unlike traditional uniform shelterwood or clear‐cut systems, ISS involves selective and spatially variable removal of mature trees, maintaining a heterogeneous canopy that mimics natural forest dynamics (Raymond and Bédard 2017; Pommerening et al. 2024; Pokhrel et al. 2024). This approach has shown potential to improve light conditions, promote regeneration, and maintain biodiversity (Löf et al. 2019; McDonald et al. 2023). Previous studies in Nepal indicate that ISS can enhance regeneration and species diversity in the Terai region when properly implemented (Awasthi et al. 2015, 2020; Pokhrel et al. 2024). However, the ecological factors influencing the regeneration success of *S. robusta* under ISS remain poorly understood, particularly in community‐managed forests (Tyagi et al. 2011).

This study investigates the regeneration dynamics of *S. robusta* under the ISS in Baijalpur Community Forest, Nepal. Specifically, it aims to: (a) analyze the origin and status of *S. robusta* regeneration under ISS, (b) assess the environmental and management factors influencing regeneration, and (c) compare regeneration status between treated and untreated stands. The findings will contribute to improving silvicultural practices and sustainable forest management strategies in community forests across Nepal, supporting both ecological integrity and local livelihoods.

## 2 Materials and Methods

### 2.1 Study Area

**FIGURE 1:**
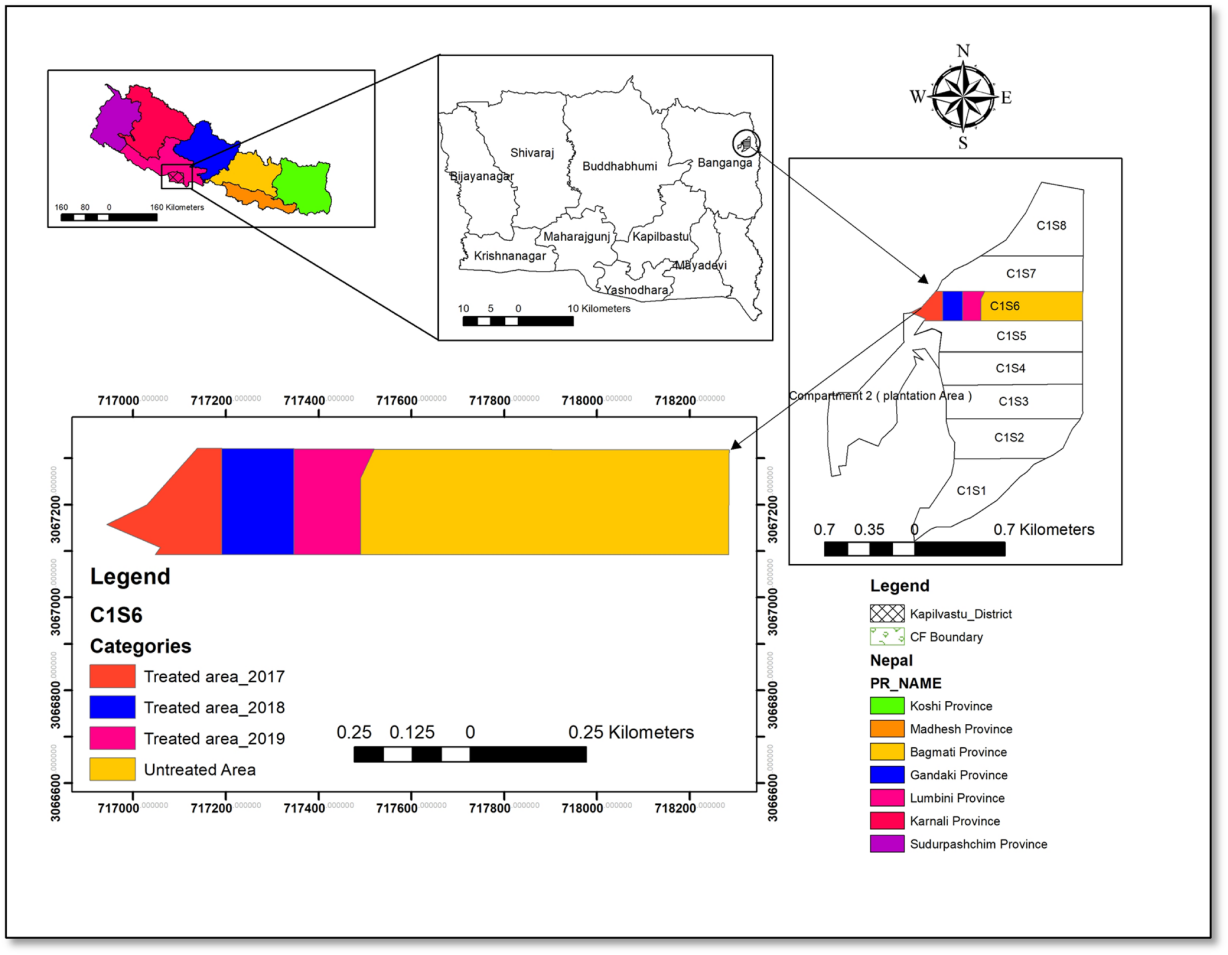
Study area map showing Baijalpur community forest in Kapilvastu district, Nepal.

The study was carried out in the Baijalpur CF, located in the Banganga Municipality‐02, Kapilvastu district, Nepal (Figure 1). Kapilvastu District lies between 93 and 1491 m above sea level and is geographically divided into the lowland plains of the Terai and the low Chure Hills. The district is located at approximately 27°32′ N latitude and 83°3′ E longitude (Bhandari 2013). The CF was registered in the Division Forest Office (DFO) Kapilvastu in 2010 and has implemented an ISS since 2017 (BCFMOP 2017). After the recent reclassification of local administrative units by the Nepal government, Baijalpur CF lies in ward no. 2 of Banganga municipality. The total area of CF is 263.43 ha where *S. robusta* is the predominant tree species. Kapilvastu experiences a humid subtropical climate with average annual temperatures ranging from 19°C to 25°C, reaching up to 43°C in summer and dropping to 4.5°C in winter. Analysis of 25 years of temperature data indicates a warming trend of 0.0216°C per year. The area receives an average annual rainfall of 1850 mm, with approximately 80% occurring during the monsoon season (mid‐June to mid‐September) (Bhandari 2013). The major soil type in the study area is sandy loam and silt clay loam (BCFMOP 2017). The forest is diversified by various flora and fauna species like *S. robusta* (Sal), *Dalbergia sissoo* (Sisau), *Terminalia bellerica* (Barro), *Teminalia tomentosa* (Saj), *Terminalia chebula* (Harro), *Syzigium cumini* (Jamun), *Adina cordifolia* (Haldu), etc. (Poudel et al. 2024). This study area falls under the WGS 1984 UTM Zone 44 N coordinates system. Five hundred and twenty‐eight household members manage the Baijalpur Community Forest User Group (BCFUG) as a user group with 3168 people. The major source of income of the people is farming, although some of them are involved in other occupations, including business and teaching (CBS 2021).

### 2.2 Irregular Shelterwood System (ISS) in the Study Area

According to the Scientific Forest Management (SFM) Guideline, 2014 and Community Forest Development Program Guideline (2014) the operational plan (OP) was prepared for 10 years from 2017 to 2027 applying the ISS as a silviculture system. *S. robusta* has a rotation age of 80 years and a regeneration period of 10 years considered a slow‐growing species (Subedi et al. 2018). However, there is no scientific evidence on the fixing of rotation age and regeneration period. The forest area was divided into two compartments, that is, compartment 1 and compartment 2. Compartment 1 was further divided into 8 sub‐compartments, each with an area of 28 ha (Figure 1). The study was conducted in sub‐compartment no. 6 (C1S6) and found to have the highest growing stock (GS) (Figure 1). Regeneration felling (RF) operation as a silviculture treatment was applied for 10 years regeneration period in this sub‐compartment. C1S5 with a GS smaller than C1S6 (28.21 ha) was selected for preparatory felling (PF) and others were selected for thinning and improvement felling operations. According to the Scientific Forest Management Guideline (2014), mother trees and felling trees were separated keeping 10–25 mother trees, to maintain the diversity of the forest. Considering the regeneration period, the C1S6 sub‐compartment was divided into 10 felling series in which yield is regulated based on a few trees. During our study, this sub‐compartment was felled in a series of 3 years that is, 2017, 2018, and 2019 which are 3 Felling series (F1, F2 and F3), which were considered as a “treated area,” whereas the remaining part of C1S6 where RF operation was not carried out was considered as “untreated area” (Figure 1). Fencing was done to protect felled areas from being disturbed and damaged by anthropogenic activities.

### 2.3 Regeneration and Soil Sample Data Collection

**FIGURE 2:**
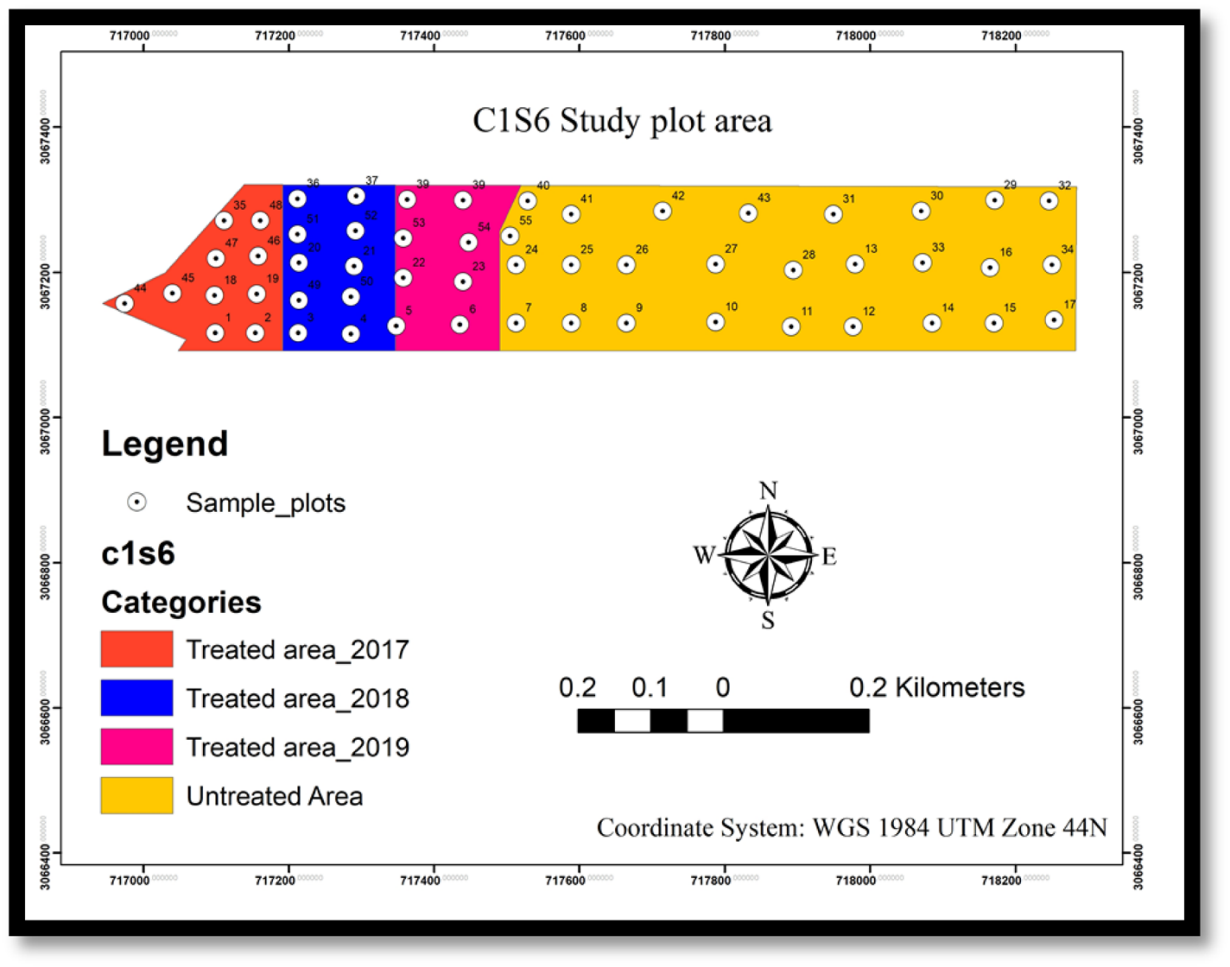
Location of sample plots in C1S6.

**FIGURE 3:**
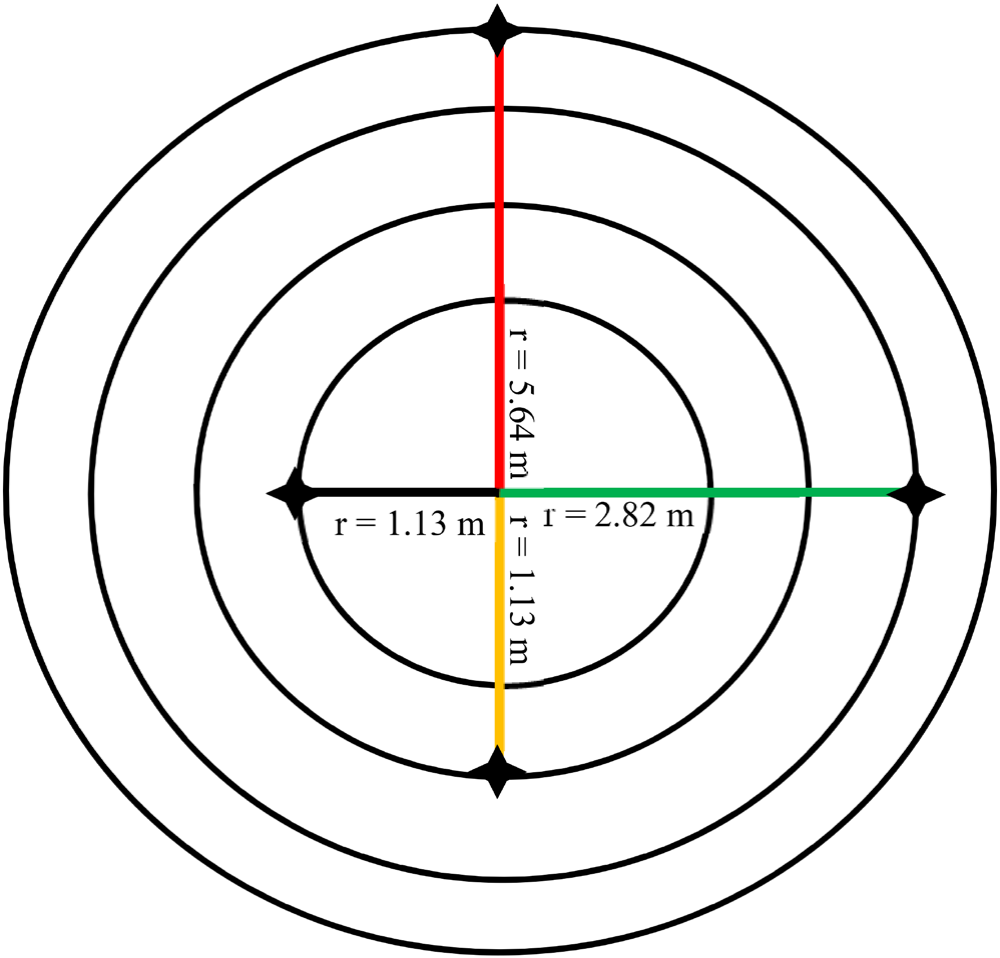
A concentric circular sample plot.

According to the Community Forest Inventory Guideline (2004) and the SFM guideline, 2014, endorsed by the Government of Nepal, a stratified systematic random sampling technique was used with a 1.5% sampling intensity, distributing 55 sample plots in the study area (Figure 2). Out of the 55 sample plots, 10 plots in 2017, 10 plots were used in 2018 and 8 plots in 2019 were used to collect data under respective years in treated areas and rest 27 plots in the untreated areas (Figure 2). Each plot was tracked using Geographic Positioning System (GPS) device where concentric plots with size 10 m × 10 m (*r* = 5.64 m) for crown cover, 5 m × 5 m (*r* = 2.82 m) for sapling, 2 m × 5 m (*r* = 1.78 m) for seedling and 2 m × 2 m (*r* = 1.13 m) for origin of seedling were used for data collection (Figure 3).

For the assessment of regeneration, the number of seedling and sapling species was counted and recorded in two phases. In the first phase, all the plots were assessed over the area while the mode of regeneration was studied through destructive sampling. Each plot was deeply investigated by digging to roots for identification of the origin of regeneration. In the second phase, only 43 plots (1–43) were enumerated in the year 2020 to study soil samples, crown cover and regeneration. While studying regeneration, the diversity of species was also covered. But the study focuses on factors affecting the regeneration of *S. robusta* . A densiometer was used to measure the crown cover from the center of plots (18 plots of treated and 25 plots of untreated area), and the measurements were averaged from four directions. While we have the felled year 2017 (F1), 2018 (F2), and 2019 (F3) in these years, the regeneration status and origin of Sal were recorded timely after the rainy season to observe the growing status. Also, the data was recorded from the fourth felling series 4 (F4). Quantitative data collected about crown cover, and origin and status of regeneration within each sample plot were tabulated in MS Excel.

The undisturbed soil samples of 500 g weight were collected from the pit dug at the center of every 43 plots using a cylindrical core having 0–20 cm depth (Bhatta and Devkota 2020) in Dec 2021. The pH, organic matter (OM), organic carbon (OC), soil texture, and NPK (Nitrogen, Phosphorus and Potassium) were recorded from the fertilizers testing laboratory at Sundarpur Kanchanpur, Nepal. They applied the following testing methods for different parameters of soil samples (Table 1).

**TABLE 1:** Soil parameter testing method in laboratory.

S. No.	Parameters	Testing methods
1	OM	Walkely‐Black method (Mylavarapu et al. 2014)
2	Nitrogen	
3	OC	
4	Phosphorus	Modified Olsens bicarbonate method (Saunders et al. 1987)
5	Potassium	Neutral ammonium acetate flame photometer method (Lu et al. 2024)
6	Texture	Hydrometer jar method (Hunduma and Kebede 2020)
7	pH	Glass electrode digital pH method (Song et al. 2020)

### 2.4 Data Analysis

Comprehensive statistical data was analyzed using R 4.0.1 software (R Studio Team 2022). Quantitative data were analyzed using descriptive statistics such as percentage, mean, frequency distribution, table, and bar plots, whereas qualitative data were analyzed descriptively. The total number of regeneration per hectare (ha) was calculated using Equation (1). Based on regeneration per ha, the condition of the forest was estimated (Table 2).

(1) $$\text{Regeneration}\;\mathrm{per}\;\mathrm{ha}=\frac{\text{Number of regeneration in each plot}}{\text{Area of sample plots}}*10,000$$

**TABLE 2:** Condition of forest regeneration based on regeneration status.

X	Condition of forest regeneration		
	Good	Medium	Poor
Seedling number	> 5000/ha	2000–5000/ha	< 2000/ha
Sapling number	> 2000/ha	800–2000/ha	< 800/ha

Various statistical tests were applied to analyze hypothetical variations in regeneration and different soil properties between treated and untreated plots. Each test served a specific purpose in evaluating the data. The Bartlett test was used to test whether multiple groups had equal variances. It checked the assumption of homogeneity of variances, which was important when applying parametric tests like ANOVA and *t*‐tests. The two‐sample *t*‐test was used to compare the means of two independent groups (e.g., treated vs. untreated seedlings and saplings) to determine if there was a statistically significant difference between the two groups. The One‐way Analysis of Variance (ANOVA) was used to compare the means of three or more groups (e.g., seedlings in different felling years) to assess if at least one group's mean is different from the others. Tukey Honest Significant Difference (HSD) test is a post hoc test performed after an ANOVA to identify which pairs of group means were significantly different. The significant difference test was carried out at a 0.05% significance level for all tests.

Soil sample results obtained from the laboratory as were interpreted as shown in Table 3; all the results were correlated with the new regeneration result as obtained from the inventory as total_reg per plot. Total reg/plot, crown cover, pH, OM, OC, nitrogen, phosphorous, potassium, and soil texture result present in the treated and untreated areas were tabulated using MS Excel for correlation analysis. Pairwise Pearson correlation analysis, among ecological variables soil indices and vegetation metrics, was carried out for the result from 55 plots and 43 plots to analyze the correlation of each soil properties effect on regeneration within the plot of study area (Benesty et al. 2009).

**TABLE 3:** Table representing the condition of different parameters of soil.

OC %	pH	OM%	Nitrogen%	Phosphorous kg/ha	Potassium kg/ha
Low: < 1%	Acidic (A): < 6.5	Low: < 2.5%	Low: < 0.1%	Low: < 31	Low: < 110
Medium: 1%–2%	Neutral (N): 6.5–7.5	Medium: 2.5%–5%	Medium: 0.1%–0.2%	Medium: 31–55	Medium: 110–280
High: > 2%	Basic (B): > 7.5	High: > 5%	High: > 0.2%	High: > 55	High: > 280

## 3 Result

### 3.1 Origin of Regeneration

**FIGURE 4:**
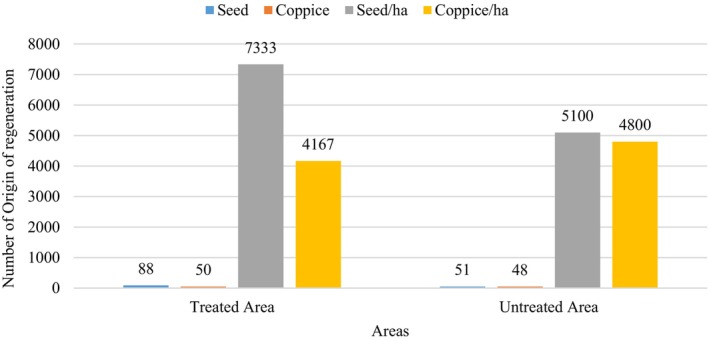
Mode of origin of Sal seedling in both treated and untreated areas.

Results from the sample plots of treated and non‐treated areas highlight that there were 139 (88 + 51) seedlings that originated from seeds, whereas 98 (50 + 48) seedlings originated from root coppice (Appendix II). The results from per ha analysis revealed that the origin of seedlings from seed was estimated to be 7333 per hectare and 5100 per hectare in treated and untreated areas (Figure 4). Similarly, there were 4167 and 4800 seedlings per ha of coppice origin in treated and untreated areas (Figure 4). A significant number of seedlings were found regenerated in treated areas than in untreated areas, while the saplings found were not well established in the plots.

### 3.2 Regeneration Status of *S. robusta* and Other Species in Treated and Untreated Areas

**FIGURE 5:**
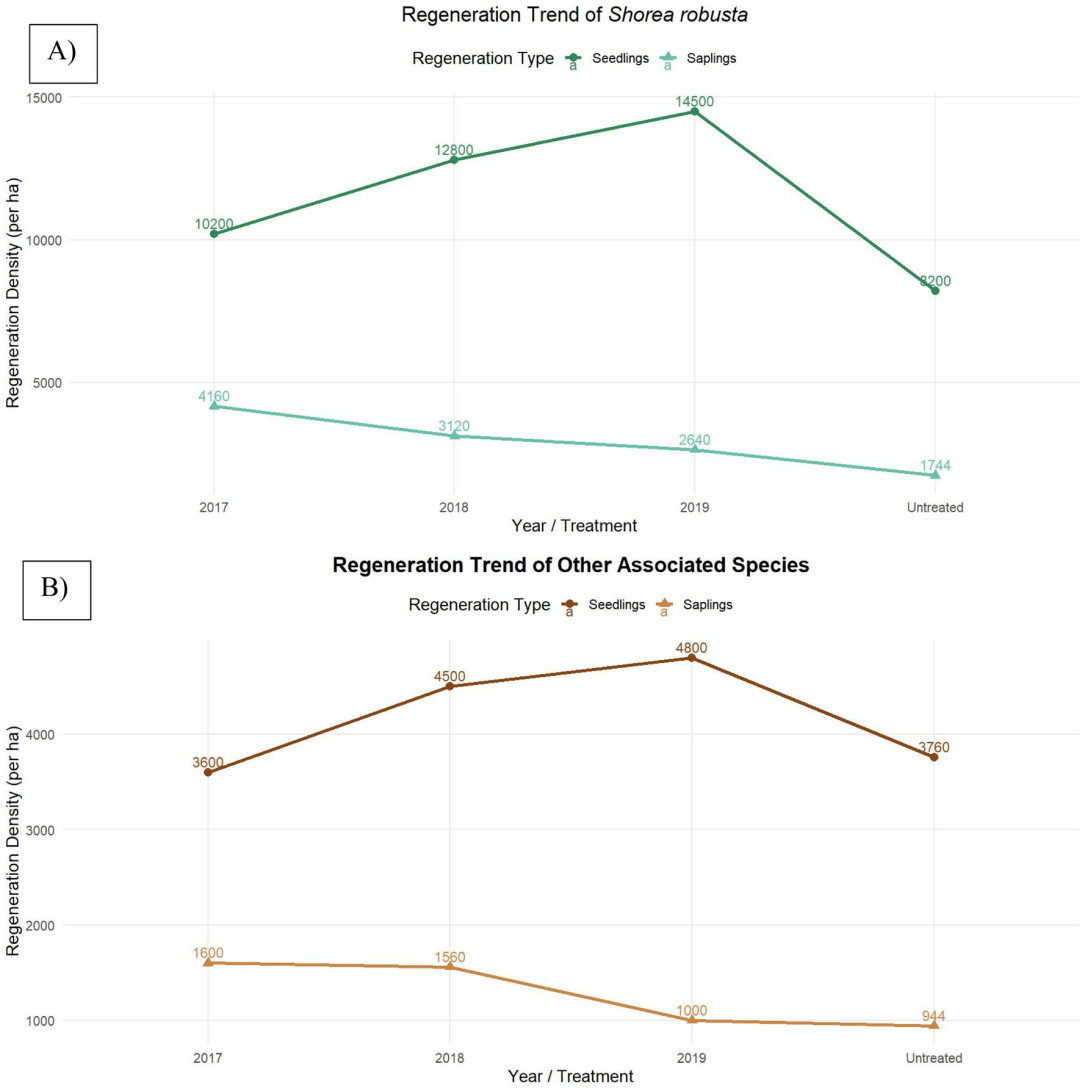
Regeneration trend of *Shorea robusta* and other associated species in treated and untreated plots.

The comparative analysis of the status of *S. robusta* regeneration within the treated areas over different felling series and untreated areas collected inside the sample plots is provided in Appendix IV. Treated areas consistently show larger numbers in per‐hectare seedling counts compared to untreated areas, with noticeable increases in seedling counts from the 2017 to 2019 felling series. There is an increasing trend of seedlings and a decreasing trend of saplings with RF applied in different felling series (Figure 5). The regeneration status of *S. robusta* species in treated areas was 623 (375 seedlings + 248 saplings) and other than Sal spp. was 233 (129 seedlings + 104 saplings). The regeneration status of *S. robusta* species in untreated area was found higher 314 (205 seedlings + 109 saplings) than other species 153 (94 seedlings + 59 saplings) (Figure 5). Treated areas outperform untreated areas in terms of both seedlings and saplings per hectare, especially when considering total regeneration ( *S. robusta* and other species combined).

According to the status of regeneration provided in Figure 5 compared with Table 2 (the condition of forest regeneration), on average, we found the *S. robusta* regeneration was in good condition within both treated and untreated areas. While the regeneration of other species was in medium condition.

#### 3.2.1 Variation Test of Seedlings and Saplings in Treated and Untreated Areas

##### 3.2.1.1 Seedlings Variation Test

Bartlett's test of homogeneity of variances indicated that the variances of the seedlings between different felling years were significantly different or not homogeneous (*K*‐squared = 14.123, *p*‐value < 0.05 at a 5% significance level). The two‐sample *t*‐test resulted in a very small *p*‐value at a 5% significance level (much less than 0.05), suggesting a significant difference in mean values of untreated and treated seedlings. The mean for treated seedlings is 11.96, and for untreated seedlings, it is 16.80.

##### 3.2.1.2 Saplings Variation Test

Bartlett's test of homogeneity of variances resulted in a *p*‐value close to 0.05, suggesting marginally significant evidence that the variances of saplings between different felling years are not homogeneous (*K*‐squared = 2.3742, *p*‐value = 0.04984 [i.e., < 0.05] at a 5% significance level). From the two‐sample *t*‐test (treated vs. untreated saplings) we found the *p*‐value is extremely small, indicating a highly significant difference of saplings between the untreated and treated areas. The mean for untreated saplings is 6.72, and for treated saplings, it is 11.40.

#### 3.2.2 Status of Seedlings and Saplings Regeneration in Three Different Treated Years

**FIGURE 6:**
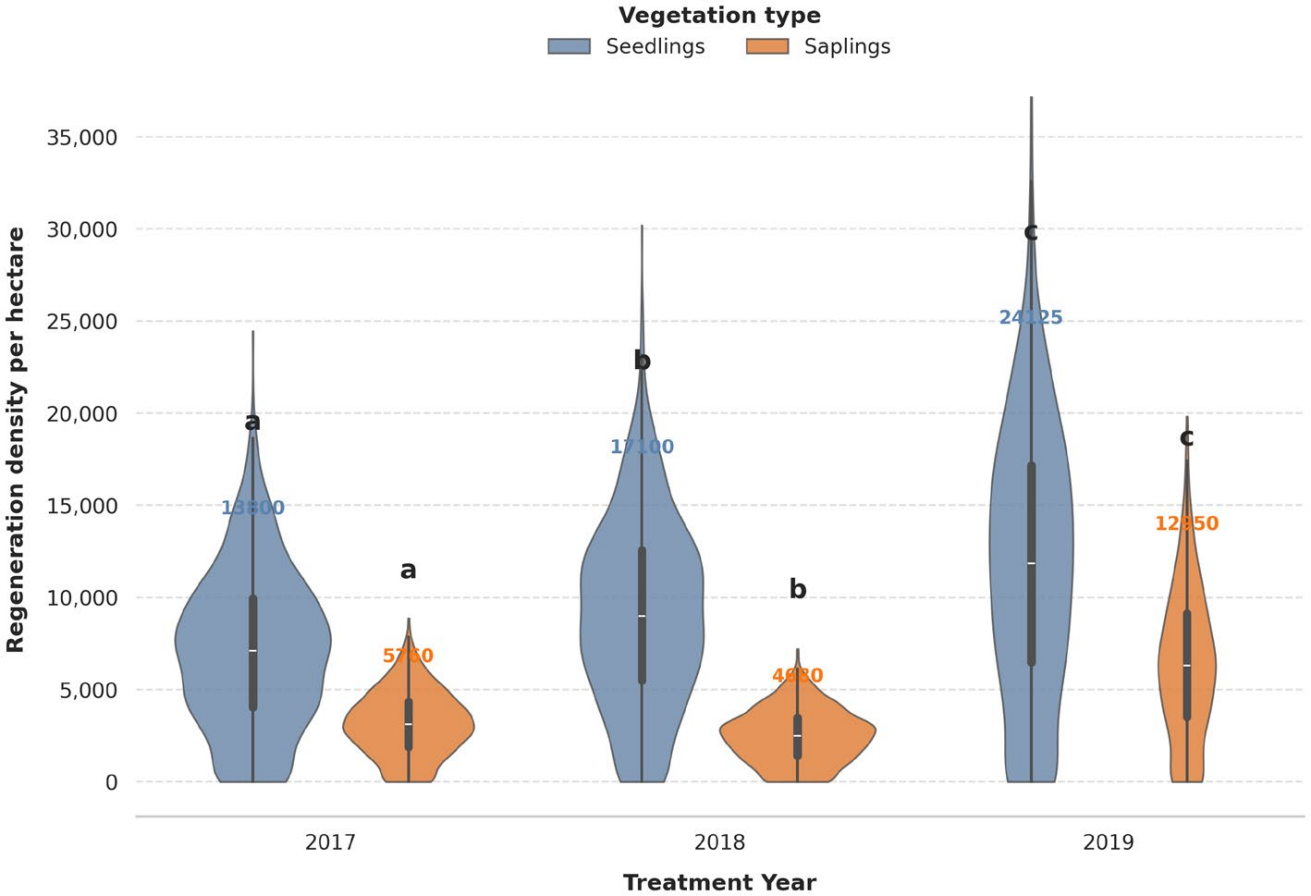
Violinplot and boxplot showing seedlings and saplings regeneration in the different treated years.

For seedlings, a one‐way ANOVA was used to test whether the mean seedling density differed among the three treatment years (2017, 2018, and 2019). The effect of year was not statistically significant (*F*_2,3_ = 0.319, *p* = 0.749), indicating that observed differences in seedling density cannot be reliably attributed to the treatment year (Figure 6). Tukey's HSD post hoc test further confirmed the absence of significant pairwise differences between years. The mean density in 2018 was 1750 seedlings ha^−1^ higher than in 2017, and 2019 showed increases of 5162.5 and 3412.5 seedlings ha^−1^ compared to 2017 and 2018, respectively, but in all cases the 95% confidence intervals included zero and adjusted *p*‐values (0.7365–0.9622) were far above 0.05.

Similarly, for saplings, one‐way ANOVA showed that the effect of year was not statistically significant (*F*_2,3_ = 1.23, *p* = 0.407), suggesting that variation in sapling density is not clearly explained by differences among the three years. Tukey's HSD pairwise comparisons also revealed no significant differences between any year pairs. Although the mean sapling density in 2019 was numerically higher than in 2017 and 2018 (by 3595 and 4135 saplings ha^−1^, respectively), the wide confidence intervals for all contrasts encompassed zero and the adjusted *p*‐values (0.4277–0.9808) remained well above the 0.05 threshold (Figure 6). Overall, both seedlings and saplings show no statistically significant temporal change, indicating broadly similar regeneration across the three treatment years.

Although the total regeneration densities for both seedlings and saplings show noticeable numerical increases from 2017 to 2019, these differences were not statistically significant (ANOVA *p* > 0.05; Tukey HSD *p* > 0.7) (Figure 6). This result is explained by two factors: (1) the high variability within each year, as shown by the wide distribution of values in the violin plots, and (2) the small sample size, which reduces statistical power. Because the within‐year variance is large relative to the between‐year differences, the confidence intervals for the pairwise comparisons overlap substantially. As a result, even large differences in mean values cannot be detected as statistically significant.

### 3.3 Factors Affecting the Regeneration Status of *S. robusta*

**FIGURE 7:**
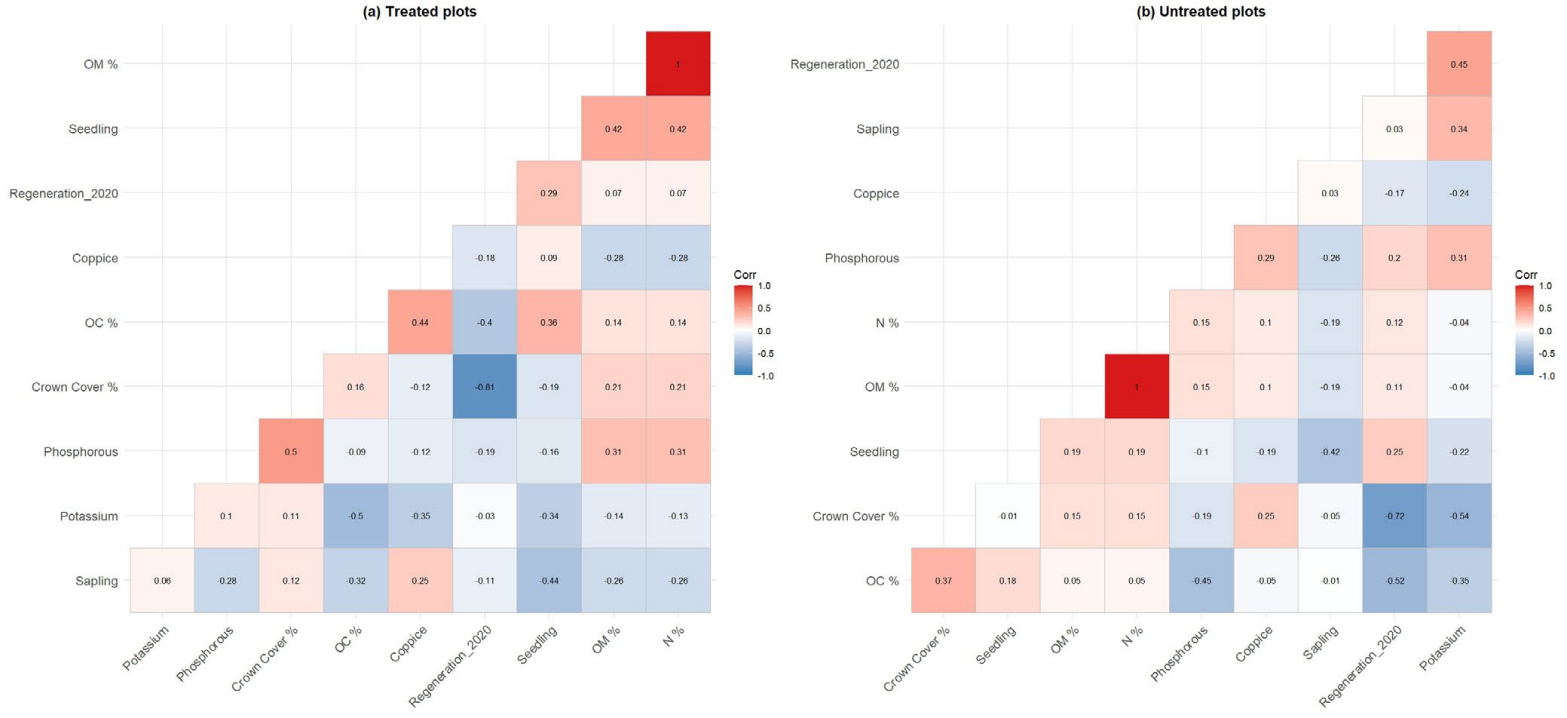
Correlation matrix heatmap for the Treated and Untreated areas showing the factors affecting the regeneration of *Shorea robusta* in the study area.

The soil test results show parameters such as pH, OM, OC, N, Phosphorous, Potassium, and soil texture within 43 sample plots of C1S6 treated and untreated areas (Appendix IV). Figure 7 indicates the conditional feature of different parameters affecting regeneration according to the values. The crown cover ranges from 11% to 90% in both areas. Treated areas were found to have higher crown cover % (up to 90%) compared to untreated plots. The pH levels range between 5.43 and 6.87 indicating a slightly acidic soil, which is common in forested areas. No significant difference in pH is observed between treated and untreated plots. The value required for soils with pH closer to neutral (6.0–6.5) tends to have a more balanced nutrient content. OM content ranges from 1.06% to 3.59%. Untreated plots tend to have lower OM levels, while treated plots have higher OM percentages (up to 3.59%). Example: Plot 5 (treated) has 3.59% OM, which is high, indicating improved fertility, compared to untreated plots like Plot 1 with 1.41% OM. OC, a key component of organic matter, ranges from 0.82% to 3.79%. Similar to OM, treated plots show higher OC compared to untreated plots, with the highest OC observed at 3.79% in Plot 11 (untreated). N content ranges from 0.05% to 0.18%. Treated plots (e.g., Plot 5, 0.18% N) tend to have higher N content than untreated plots. P content ranges from 6.15 to 347.45 kg/ha. Plot 12 (untreated) shows an extremely high P content (347.45 kg/ha). In contrast, treated plots like Plot 10 have much lower P (8.86 kg/ha).

There is no clear correlation between treatment and K levels. Plot 11 (untreated) has the highest K level (361.2 kg/ha), while Plot 10 (treated) has a much lower K level (102 kg/ha). Treated area soils do not necessarily show higher K content. Sandy loam appears to be the most common soil type in the sample. Treated plots are found across all soil types, indicating that treatment is applied across varying textures. Sandy loam soils are common in both treated and untreated areas, but treated silty clay loam soils like Plots 4 and 5 tend to have better nutrient retention (Appendix IV). The result revealed that the three variables crown cover, OM, and OC had significant effects on regeneration.

In the treated area, moderate positive correlations were observed between seedling and sapling densities (*r* = 0.62) and between coppice and recent regeneration (Regeneration_2020; *r* = 0.48). Additionally, OC showed a moderate association with crown cover (*r* = 0.45), indicating that canopy recovery may be contributing to soil carbon accumulation. In contrast, the untreated area exhibited stronger correlations across several ecological variables, notably between coppice and Regeneration_2020 (*r* = 0.85), and between P and K (*r* = 0.69). The untreated matrix also showed a robust relationship between OM and OC (*r* = 0.63) (Figure 7). While seedling–sapling correlations remained consistent across both matrices (*r* = 0.62), the overall pattern suggests that treated areas are characterized by managed regeneration and canopy‐driven soil recovery, whereas untreated areas maintain stronger internal ecological linkages driven by self‐organizing processes.

## 4 Discussion

This study shows that the ISS applied in Baijalpur Community Forest has enhanced the regeneration of *S. robusta* , mainly by promoting seed‐origin seedlings in treated stands compared to untreated areas (Figure 4). The higher proportion and density of seedlings from seed in treated plots suggest that canopy opening, reduced competition, and improved light conditions have created more favorable micro‐sites for germination and early establishment, consistent with the light‐demanding nature of *S. robusta* reported in previous studies (Khadka et al. 2023; Pokhrel et al. 2024). At the same time, a substantial share of regeneration originated from coppice, which reflects the species' capacity to resprout following disturbance and the die‐back phenomenon described for *S. robusta* and similar species such as *Dalbergia sissoo* (Rautiainen and Suoheimo 1997; Shah et al. 2021).

Although seedling and sapling densities were consistently higher in treated than untreated areas, the ANOVA and Tukey HSD results indicated no statistically significant differences among the three felling years for either seedlings or saplings (Appendix III and Figure 6). This apparent mismatch between numerical trends and statistical significance can be explained by high within‐year variability and small sample size, which reduce the power to detect treatment‐year effects. Nevertheless, the overall pattern‐higher total regeneration under ISS and good regeneration status according to national guidelines‐supports the conclusion that ISS is effective in promoting *S. robusta* regeneration when combined with protection measures such as fencing, cleaning, and weeding. Protection of theses sepcies from fire and grazing is essential for regeneration (Malla and Acharya 2018). The declining sapling densities in more recently treated coupes suggest that it may take several years for seedlings to transition to saplings, as also noted by Shrestha et al. (2019) and Aryal et al. (2021), highlighting the need for longer‐term monitoring of cohort development under ISS.

Soil conditions in the study area generally matched the ecological requirements of *S. robusta* , with slightly acidic to near‐neutral pH and predominantly sandy loam textures (Gautam and Devoe 2006). Treated plots tended to show improved organic matter, nitrogen, and organic carbon levels, indicating that management interventions under ISS can contribute to gradual soil recovery and fertility enhancement. However, regeneration performance was not clearly differentiated along soil nutrient gradients, and some untreated plots exhibited locally high phosphorus or potassium values. Correlation analysis further revealed that crown cover, organic matter, and organic carbon were the key variables significantly associated with regeneration, whereas N, P, and K played a less distinct role within the range of conditions observed (Figure 7). The negative relationship between crown cover and regeneration supports the idea that moderate canopy opening is critical for *S. robusta* recruitment and is consistent with other studies reporting reduced regeneration under dense, unmanaged canopy (Baral and Ghimire 2020; Poudel and Devkota 2021).

The contrasting correlation patterns between treated and untreated plots suggest different mechanisms structuring stand dynamics. In treated plots, moderate positive correlations between seedling and sapling densities and between coppice and recent regeneration point toward a regeneration layer increasingly shaped by planned interventions and canopy manipulation. The positive association between crown cover and organic carbon in treated areas indicates that as the canopy recovers after felling, litter inputs and soil carbon accumulation increase, reinforcing soil quality. In contrast, untreated plots showed stronger internal linkages between coppice and recent regeneration and between phosphorus and potassium, reflecting self‐organized nutrient cycling and successional processes in the absence of active management. Together, these findings indicate that ISS not only alters stand structure and light regimes but also gradually reshapes soil‐vegetation interactions in ways that can support long‐term forest resilience if carefully regulated.

This study has several limitations that should be considered when interpreting the results. First, it was conducted in a single community forest with relatively similar edaphic and climatic conditions, which constrains the generalization of the findings to other regions. Second, the analysis covers only the first three felling series (2017–2019), which may not fully capture longer‐term trajectories of seedling and sapling survival, especially given the high early mortality often observed in tropical forests (Chazdon and Guariguata 2016). Third, while we included a range of soil parameters, other potentially important factors, such as microtopography, herbaceous competition, grazing intensity, and fire history, were not explicitly quantified and may partly explain residual variation in regeneration.

Despite these constraints, the study provides important empirical evidence in support of ISS as a silvicultural system capable of enhancing natural regeneration of *S. robusta* in community‐managed forests of the Terai. By combining moderate canopy opening, retention of mother trees, and protection of regeneration, ISS can help address long‐standing concerns about regeneration failure under conventional community forestry practices. Going forward, long‐term, multisite studies that integrate detailed soil, microclimatic, and disturbance data are needed to refine ISS prescriptions, including optimal canopy retention levels, felling cycles, and post‐harvest tending regimes. Strengthening the linkage between research and community‐based management through adaptive monitoring, local capacity building, and participatory decision‐making will be essential to ensure that ISS contributes to both ecological integrity and the livelihoods of forest‐dependent households, as envisioned in Nepal's sustainable forest management agenda.

## 5 Conclusion

The study demonstrates that the ISS effectively enhances the regeneration of *S. robusta* in Baijalpur Community Forest by increasing both seedling and sapling densities in managed areas. The positive influence of ISS on regeneration highlights its potential as a valuable tool for sustainable forest management in Nepal's community forestry context. While treated areas exhibited better soil quality, organic matter, and nutrient conditions conducive to growth, no significant variation in regeneration was attributed solely to soil nutrients, indicating that other environmental and management factors—particularly canopy structure and organic carbon levels—play critical roles.

However, the study's scope was limited to a single forest and a short monitoring period, suggesting the need for long‐term, multisite research to validate these findings under varied ecological conditions. Future efforts should integrate comprehensive soil and microclimatic analyses, ensure continuous monitoring, and actively involve local communities in adaptive forest management. Promoting both seedling and coppice regeneration will strengthen genetic diversity and forest resilience. Overall, the ISS represents a promising, ecologically sound approach to restoring and maintaining healthy *S. robusta* forests in Nepal's lowland regions.

## Author Contributions

**Sudhan Gaire:** conceptualization (lead), data curation (equal), formal analysis (lead), investigation (lead), methodology (lead), resources (lead), software (equal), supervision (equal), validation (equal), writing – original draft (lead). **Sandesh Gharti:** data curation (equal), formal analysis (equal), resources (equal), supervision (equal), validation (equal), visualization (equal), writing – original draft (equal), writing – review and editing (equal). **Rohit Bhusal:** data curation (supporting), formal analysis (supporting), software (supporting), validation (supporting). **Badri Bhattarai:** writing – review and editing (equal). **Sachin Timilsina:** formal analysis (supporting), visualization (supporting), writing – review and editing (equal). **Dipendra Dhungana:** formal analysis (supporting), software (supporting), visualization (supporting), writing – review and editing (supporting).

## Funding

The authors have nothing to report.

## Disclosure

Open Access: This article is licensed under a Creative Commons Attribution 4.0 International License, which permits the use, sharing, adaptation, distribution, and reproduction in any medium or format, as long as you give appropriate credit to the original author(s) and the source, provide a link to the Creative Commons license, and indicate if changes were made. The images or other third‐party materials in this article are included in the article's Creative Commons license unless indicated otherwise in a credit line to the material. If the material is not included in the article's Creative Commons license and your intended use is not permitted by statutory regulation or exceeds the permitted use, you will need to obtain permission directly from the copyright holder. To view a copy of this license, visit http://creativecommons.org/licenses/by/4.0/.

## Consent

The publishing of this research article has the complete approval of all its authors. We all concur that the material, conclusions, and submission for publication are sound.

## Conflicts of Interest

The authors declare no conflicts of interest.

## Supporting information


**Data S1:** ece372885‐sup‐0001‐Supinfo01.docx.

## Data Availability

All the required data are uploaded as Supporting Information.
