# Correction to “Effects of the Irregular Shelterwood System on Regeneration Dynamics of *Shorea robusta* Gaertn. f. in Baijalpur Community Forest, Nepal”

**DOI:** 10.1002/ece3.73136

**Published:** 2026-02-17

Gaire, S., S. Gharti, R. Bhusal, B. Bhattarai, S. Timilsina, D. Dhungana. 2026. “Effects of the Irregular Shelterwood System on Regeneration Dynamics of *Shorea robusta* Gaertn. f. in Baijalpur Community Forest.” *Nepal Ecology and Evolution* 16, no. 1: e72885. https://doi.org/10.1002/ece3.72885.

Figure 3 in the published article is incorrect. The corrected Figure 3 is shown below.

**FIGURE 3:**
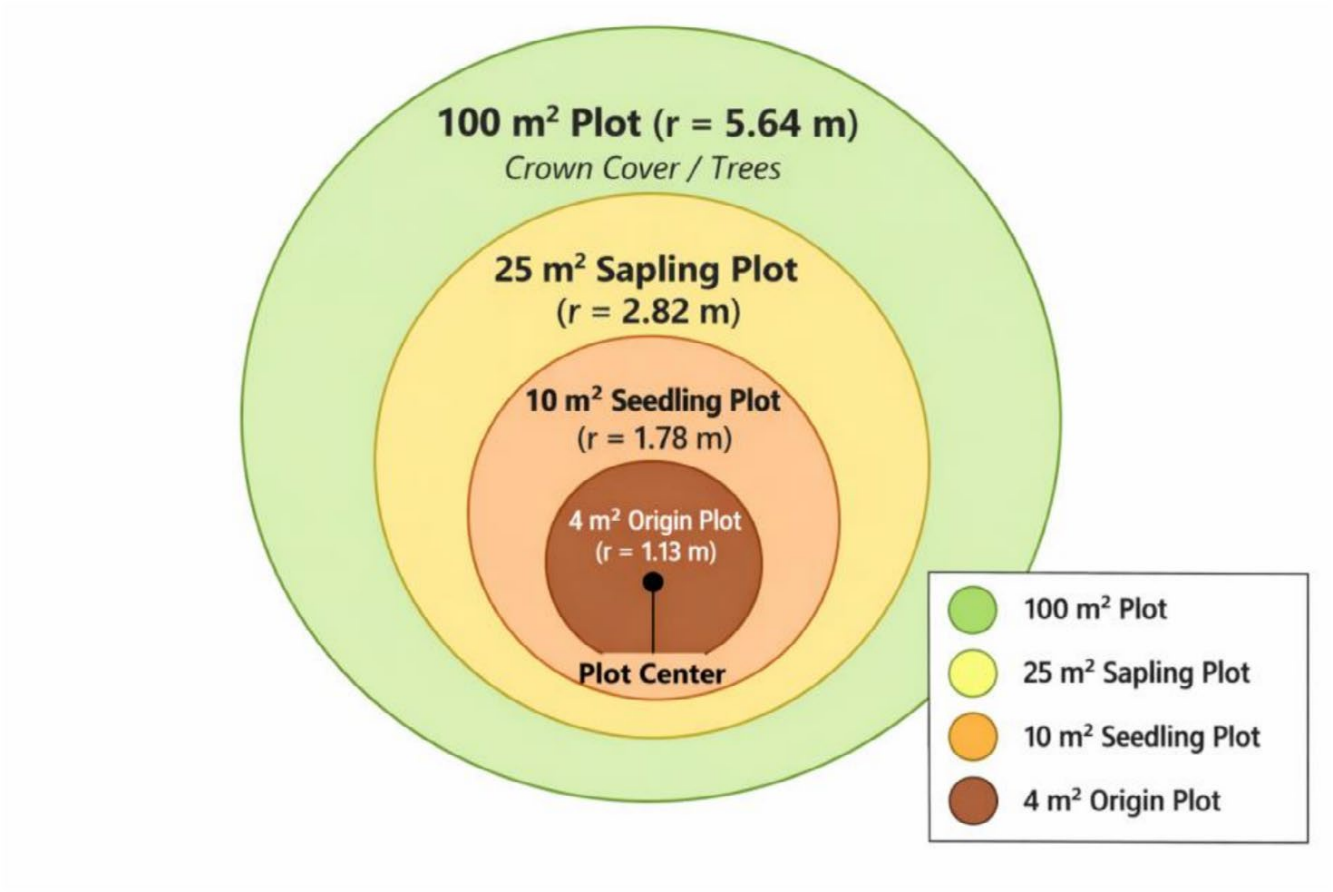
A concentric circular plot.

We apologize for this error.

