# Supplementary material for: Effects of the Irregular Shelterwood System on Regeneration Dynamics of Shorea robusta Gaertn. f. in Baijalpur Community Forest, Nepal
Source: Ecol Evol. 2026 Jan 4;16(1):e72885. doi: 10.1002/ece3.72885 (PMC12765655; doi:10.1002/ece3.72885)
Supplement: Supplementary file 1 — Data S1: ece372885‐sup‐0001‐Supinfo01.docx. [file ECE3-16-e72885-s001.docx]

**Supplementary Files:**

**Appendix I: Photos Plates of research work**

| 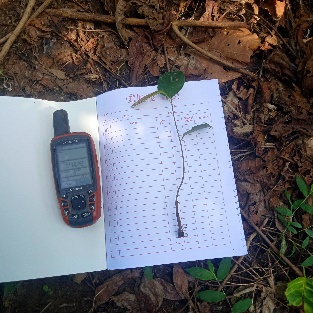  Seedlings origin identification | 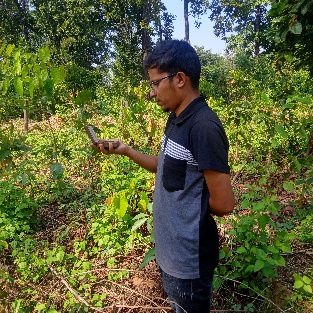  Tracking out plots |
| --- | --- |
| 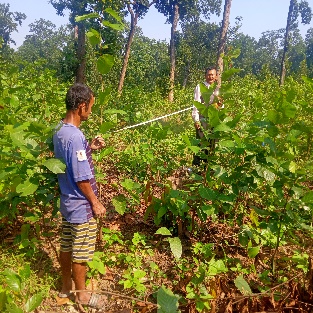  Assessing the plot | 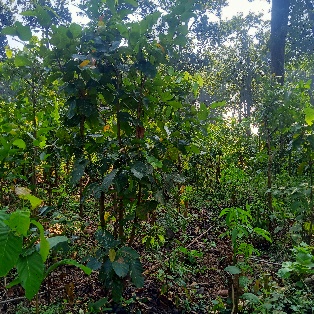  Sal in the forest |
| 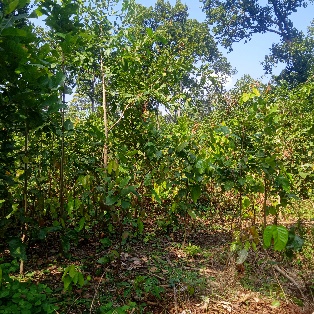  Seedlings and sapling in the plot | 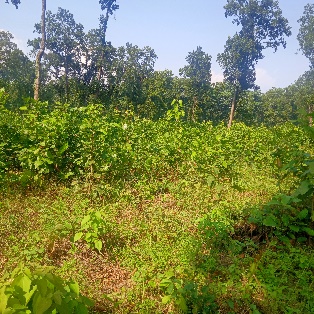  Seedlings and sapling in the plot |

**Appendix II: Status of regeneration in treated and untreated areas of C1S6**

| **Regeneration status of *S. robusta*** | | | | | |
| --- | --- | --- | --- | --- | --- |
| Treated Year | Seedlings | | Saplings | Per ha Seedlings | Per ha saplings |
| 2017 | 102 | | 104 | 10200 | 4160 |
| 2018 | 128 | | 78 | 12800 | 3120 |
| 2019 | 145 | | 66 | 14500 | 2640 |
| Untreated | 205 | | 109 | 8200 | 1744 |
| **Regeneration status of other than *S. robusta*** | | | | | |
| Treated Year | Seedlings | | Saplings | Per ha Seedlings | Per ha saplings |
| 2017 | 36 | | 40 | 3600 | 1600 |
| 2018 | 45 | | 39 | 4500 | 1560 |
| 2019 | 48 | | 25 | 4800 | 1000 |
| Untreated | 94 | | 59 | 3760 | 944 |
| **Total no. of Regeneration** | | | | | |
| Treated Year | Seedlings | | Saplings | Per ha Seedlings | Per ha saplings |
| 2017 | 138 | | 144 | 13800 | 5760 |
| 2018 | 173 | | 117 | 17300 | 4680 |
| 2019 | 193 | | 91 | 19300 | 3640 |
| Untreated | 299 | | 168 | 11960 | 2688 |
| **Average number of Regeneration** | | | | | |
| Area | | Per ha seedlings | | Per ha Saplings | |
| Treated | | 16800 | | 4693 | |
| Untreated | | 11960 | | 2688 | |

**Appendix III Seedling and Saplings ANOVA summary and Tukey HSD pairwise comparisons**

**1. Seedlings – ANOVA Summary**

| **Source** | **Df** | **Sum Sq** | **Mean Sq** | **F value** | **Pr(>F)** |
| --- | --- | --- | --- | --- | --- |
| Year | 2 | 27,572,708 | 13,786,354 | 0.319 | 0.749 |
| Residuals | 3 | 129,732,812 | 43,244,271 | — | — |

**Seedlings – Tukey HSD Pairwise Comparisons**

**Tukey multiple comparisons of means**

**95% family-wise confidence level**

| **Comparison** | **diff** | **lwr** | **upr** | **p adj** |
| --- | --- | --- | --- | --- |
| 2018 – 2017 | 1750.0 | -25,729.7 | 29,229.7 | 0.9622 |
| 2019 – 2017 | 5162.5 | -22,317.2 | 32,642.2 | 0.7365 |
| 2019 – 2018 | 3412.5 | -24,067.2 | 30,892.2 | 0.8681 |

**2. Saplings – ANOVA Summary**

| **Source** | **Df** | **Sum Sq** | **Mean Sq** | **F value** | **Pr(>F)** |
| --- | --- | --- | --- | --- | --- |
| Year | 2 | 20,209,233 | 10,104,617 | 1.23 | 0.407 |
| Residuals | 3 | 24,654,850 | 8,218,283 | — | — |

**Saplings – Tukey HSD Pairwise Comparisons**

**Tukey multiple comparisons of means**

**95% family-wise confidence level**

| **Comparison** | **diff** | **lwr** | **upr** | **p adj** |
| --- | --- | --- | --- | --- |
| 2018 – 2017 | -540 | -12,519.49 | 11,439.49 | 0.9808 |
| 2019 – 2017 | 3595 | -8,384.49 | 15,574.49 | 0.5054 |
| 2019 – 2018 | 4135 | -7,844.49 | 16,114.49 | 0.4277 |

**Appendix IV: Value of the different parameters obtained from soil test of different plots**

| **Plot No.** | **Total reg/ plot_2020** | **Crown cover %** | **PH** | **Type** | **OM %** | **OC %** | **N%** | **P2O5 Kg/ha** | **K2O Kg/ha** | **Texture** | Area |
| --- | --- | --- | --- | --- | --- | --- | --- | --- | --- | --- | --- |
| 1 | 31 | 15 | 6.41 | A | 1.41 | 0.82 | 0.07 | 13.85 | 81.60 | Sandy Loam | Treated_2017 |
| 2 | 29 | 16 | 6.53 | N | 2.58 | 1.50 | 0.13 | 21.82 | 151.20 | Silty loam | Treated_2017 |
| 3 | 32 | 20 | 6.28 | A | 2.34 | 3.64 | 0.12 | 38.68 | 82.80 | Silty clay loam | Treated_2018 |
| 4 | 28 | 13 | 6.34 | A | 1.92 | 3.68 | 0.10 | 63.07 | 70.80 | Silty clay loam | Treated_2018 |
| 5 | 31 | 12 | 6.16 | A | 3.59 | 3.57 | 0.18 | 45.00 | 78.00 | Silty clay loam | Treated_2019 |
| 6 | 31 | 18 | 5.96 | N | 1.96 | 1.14 | 0.10 | 13.85 | 175.20 | Silty loam | Treated_2019 |
| 7 | 34 | 14 | 6.43 | A | 1.90 | 1.10 | 0.09 | 140.23 | 175.20 | Silty clay loam | Untreated |
| 8 | 27 | 17 | 5.91 | A | 2.67 | 3.43 | 0.13 | 112.77 | 49.20 | Silty clay loam | Untreated |
| 9 | 32 | 15 | 6.29 | A | 1.39 | 3.65 | 0.07 | 64.88 | 116.40 | Silty clay loam | Untreated |
| 10 | 30 | 12 | 6.28 | A | 2.77 | 3.64 | 0.14 | 8.86 | 102.00 | Silty clay loam | Untreated |
| 11 | 28 | 11 | 6.53 | A | 2.44 | 3.79 | 0.12 | 66.69 | 361.20 | Sandy Loam | Untreated |
| 12 | 30 | 13 | 6.14 | A | 2.12 | 1.23 | 0.11 | 347.45 | 228.00 | Sandy Loam | Untreated |
| 13 | 43 | 22 | 6.87 | A | 2.35 | 1.36 | 0.12 | 55.97 | 165.60 | Sandy Loam | Untreated |
| 14 | 24 | 21 | 6.10 | A | 1.46 | 3.54 | 0.07 | 35.97 | 34.80 | Sandy Loam | Untreated |
| 15 | 29 | 13 | 6.25 | A | 1.59 | 3.63 | 0.08 | 73.01 | 111.60 | Sandy Loam | Untreated |
| 16 | 29 | 14 | 5.99 | A | 2.11 | 3.47 | 0.11 | 97.41 | 123.60 | Sandy Loam | Untreated |
| 17 | 31 | 12 | 5.95 | A | 2.11 | 3.45 | 0.11 | 139.88 | 188.40 | Sandy Loam | Untreated |
| 18 | 26 | 17 | 5.88 | A | 2.15 | 3.41 | 0.11 | 37.77 | 104.40 | Sandy Loam | Treated_2017 |
| 19 | 21 | 76 | 6.43 | A | 2.97 | 1.73 | 0.15 | 26.37 | 110.40 | Sandy Loam | Treated_2017 |
| 20 | 26 | 70 | 5.98 | A | 2.84 | 1.65 | 0.14 | 104.93 | 228.00 | Sandy Loam | Treated_2018 |
| 21 | 22 | 80 | 5.62 | A | 1.99 | 1.16 | 0.10 | 155.03 | 163.20 | Sandy Loam | Treated_2018 |
| 22 | 25 | 85 | 5.43 | A | 3.10 | 1.80 | 0.16 | 122.01 | 141.60 | Sandy Loam | Treated_2019 |
| 23 | 24 | 90 | 6.15 | A | 2.90 | 3.57 | 0.14 | 67.59 | 46.80 | Sandy Loam | Treated_2019 |
| 24 | 25 | 85 | 6.27 | A | 2.70 | 3.64 | 0.14 | 215.79 | 68.40 | Sandy Loam | Untreated |
| 25 | 28 | 70 | 5.93 | A | 2.70 | 3.44 | 0.14 | 50.42 | 46.80 | Sandy Loam | Untreated |
| 26 | 24 | 72 | 6.20 | A | 2.15 | 3.60 | 0.11 | 83.86 | 49.20 | Sandy soil | Untreated |
| 27 | 29 | 80 | 5.78 | A | 2.44 | 3.35 | 0.12 | 109.16 | 63.60 | Sandy Loam | Untreated |
| 28 | 26 | 84 | 6.23 | A | 3.13 | 3.61 | 0.16 | 148.92 | 102.00 | Sandy Loam | Untreated |
| 29 | 23 | 83 | 6.42 | A | 1.88 | 3.72 | 0.09 | 67.59 | 135.60 | Sandy Loam | Untreated |
| 30 | 21 | 82 | 6.20 | A | 2.77 | 3.60 | 0.14 | 17.89 | 44.40 | Sandy Loam | Untreated |
| 31 | 21 | 76 | 6.11 | A | 2.31 | 3.54 | 0.12 | 67.59 | 126.00 | Sandy Loam | Untreated |
| 32 | 16 | 88 | 6.08 | A | 1.06 | 3.53 | 0.05 | 49.52 | 106.80 | Sandy soil | Untreated |
| 33 | 12 | 76 | 6.14 | A | 1.49 | 3.56 | 0.07 | 103.74 | 66.00 | Sandy Loam | Untreated |
| 34 | 13 | 89 | 6.07 | A | 2.74 | 3.52 | 0.14 | 151.63 | 56.40 | Sandy Loam | Untreated |
| 35 | 15 | 87 | 6.29 | A | 1.82 | 3.65 | 0.09 | 44.10 | 85.20 | Sandy Loam | Treated_2017 |
| 36 | 21 | 86 | 6.15 | A | 2.97 | 3.57 | 0.15 | 82.95 | 92.40 | Sandy Loam | Treated_2018 |
| 37 | 16 | 80 | 6.45 | N | 1.72 | 3.74 | 0.09 | 33.25 | 193.20 | Sandy soil | Treated_2018 |
| 38 | 15 | 76 | 6.33 | A | 2.24 | 3.67 | 0.11 | 6.15 | 126.00 | Sandy soil | Treated_2019 |
| 39 | 16 | 84 | 6.21 | A | 2.80 | 3.60 | 0.14 | 123.62 | 63.60 | Sandy soil | Treated_2019 |
| 40 | 18 | 83 | 6.11 | A | 2.05 | 3.54 | 0.10 | 38.68 | 87.60 | Sandy Loam | Untreated |
| 41 | 22 | 82 | 5.77 | A | 1.75 | 3.35 | 0.09 | 13.37 | 68.40 | Sandy Loam | Untreated |
| 42 | 17 | 79 | 5.90 | A | 2.57 | 3.42 | 0.13 | 22.41 | 85.20 | Sandy Loam | Untreated |
| 43 | 15 | 90 | 5.78 | A | 2.11 | 3.35 | 0.11 | 13.37 | 30.00 | Sandy soil | Untreated |
